# An Exploration of Machine Learning Methods for Robust Boredom Classification Using EEG and GSR Data

**DOI:** 10.3390/s19204561

**Published:** 2019-10-20

**Authors:** Jungryul Seo, Teemu H. Laine, Kyung-Ah Sohn

**Affiliations:** 1 Department of Computer Engineering, Ajou University, Suwon 16499, Korea; jrseojr@naver.com; 2 Department of Computer Science, Electrical and Space Engineering, The Luleå University of Technology, Skellefteå 93187, Sweden; teemu@ubilife.net

**Keywords:** boredom, machine learning, emotion, EEG, GSR, classification, sensor

## Abstract

In recent years, affective computing has been actively researched to provide a higher level of emotion-awareness. Numerous studies have been conducted to detect the user’s emotions from physiological data. Among a myriad of target emotions, boredom, in particular, has been suggested to cause not only medical issues but also challenges in various facets of daily life. However, to the best of our knowledge, no previous studies have used electroencephalography (EEG) and galvanic skin response (GSR) together for boredom classification, although these data have potential features for emotion classification. To investigate the combined effect of these features on boredom classification, we collected EEG and GSR data from 28 participants using off-the-shelf sensors. During data acquisition, we used a set of stimuli comprising a video clip designed to elicit boredom and two other video clips of entertaining content. The collected samples were labeled based on the participants’ questionnaire-based testimonies on experienced boredom levels. Using the collected data, we initially trained 30 models with 19 machine learning algorithms and selected the top three candidate classifiers. After tuning the hyperparameters, we validated the final models through 1000 iterations of 10-fold cross validation to increase the robustness of the test results. Our results indicated that a Multilayer Perceptron model performed the best with a mean accuracy of 79.98% (AUC: 0.781). It also revealed the correlation between boredom and the combined features of EEG and GSR. These results can be useful for building accurate affective computing systems and understanding the physiological properties of boredom.

## 1. Introduction

Sensors, machine learning, artificial intelligence, and other kinds of information technologies have recently been advancing rapidly. Based on these trends, several studies have been conducted on the acquisition and processing of information, aiming at a higher-level understanding of the collected information. In particular, detection of the user’s context, such as their emotional or physical state, is of particular importance because it enables the creation of context-aware systems that adapt their behavior to match the context in which they are used. This branch of computer science is known as context-aware computing. A sub-branch of it, which is the focus of this study, concentrates on classifying emotions using physiological data.

The study of emotion classification belongs to the area of affective computing (AC) that aims to build computer systems capable of detecting and reacting to the user’s emotions. The area of AC in computer science is considered to have been established when a seminal paper by Picard [1] was published, and it has since become a vibrant field of study, with some example studies being [2,3,4]. To classify emotions in these systems, researchers have three data collection (i.e., measurement) strategies at their disposal [5]: (i) neurological/physiological measurement, which uses sensors to detect changes in the user’s body; (ii) subjective self-reporting by questionnaires, diaries or interviews; and (iii) behavioral measurement that is based on expert observations of the participant’s behavior. While all these approaches have their specific advantages and disadvantages, as Kim and Fesenmaier [5] suggest, physiological measurement is considered to be particularly objective. In our literature review, we found that a large body of AC studies exists on classifying emotions from physiological data such as electroencephalography (EEG) [2,6,7], galvanic skin response (GSR) [2,8,9,10,11], heart rate [2,10,11], and others [12,13]. However, a higher validity can be achieved by combining more than one measurement strategy. For example, a viable approach, which is employed in this study, is to combine a physiological approach with self-reporting, where the latter is used to verify the existence of the target emotion.

Accurate classification of boredom can be considered of particular importance because boredom affects multiple facets of our lives. In a technical report published by the United States Air Force, unmanned aerial vehicle pilots’ reaction times were longer when they felt bored [14]. Furthermore, boredom can contribute to serious medical issues such as cardiovascular disease [15]. Additionally, it can have negative effects on learning [16,17,18,19]. If computing devices could accurately classify the occurrence of the user’s boredom and administer a suitable intervention to compensate for it, they could be used to tackle the aforementioned boredom-related issues.

Several previous studies have built boredom classification models using different physiological data as summarized in Table 1. However, to the best of our knowledge, no previous studies have used both EEG and GSR data for boredom classification. In this study, we performed a joint analysis of both data by collecting EEG and GSR data from 28 participants who also answered a questionnaire surveying their perceived level of boredom. The participants watched two types of video stimuli that were prepared to elicit boredom and to entertain, respectively. Based on the collected data and questionnaire results, we ran an initial test of 19 machine learning algorithms and selected the best three candidate classification models. After hyperparameter tuning, we measured the final performance of the selected models with 1000 iterations of 10-fold cross validation. The best performance with a mean accuracy of 79.98% (min: 71.43%, max: 93.93%) was obtained using a Multilayer perceptron (MLP) model. Furthermore, we analyzed the used features to investigate the correlation between EEG data, GSR data, and boredom. This study, therefore, has three major contributions: (i) revealing a correlation between EEG, GSR, and boredom; (ii) conducting a reliable performance comparison among 19 machine learning algorithms through repeated cross validations; and (iii) proposing a robust boredom classification model based on MLP.

## 2. Background

Emotion detection based on physiological data are a vibrant research field that has produced a large body of studies focusing on different emotions and analysis approaches. This section provides an overview of the previous physiology-based emotion detection research relevant to our study. In our literature review, we searched previous studies in Google Scholar with searching keywords of “Emotion”, “Boredom”, “Classification”, “Physiology”, and “Sensor”. We did not set limitations on the publication year or forum; however, we excluded studies that classified emotions solely by interviews or surveys.

**Figure 1:**
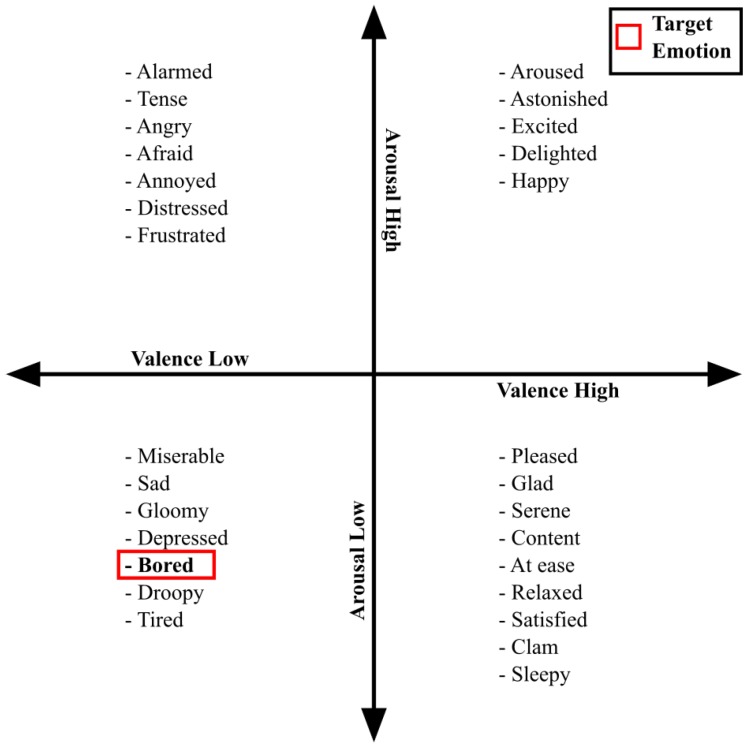
Circumplex model [26].

Several AC studies focusing on emotion classification from physiological data [2,6,20,21,22,23,24,25] referred to Russell’s Circumplex model (Figure 1) to explain the target emotion [26]. The model categorizes emotions into four groups by the dimensions of valance and arousal. According to the model, boredom is categorized into the low-valence low-arousal group (red box in Figure 1).

Despite the simplicity of the way in which the Circumplex model assigns boredom to the third quadrant, boredom is considered a complex emotion as various studies have defined it differently. Vogel-Walcutt et al.’s literature review resulted in 37 definitions of boredom, and concluded that “boredom occurs when an individual experiences both the neurological state of low arousal and the psychological state of dissatisfaction, frustration, or disinterest in response to the low arousal.” [27]. The conclusions of Russell’s model [26] and Vogel-Walcutt et al.’s definition [27] are thus similar. In contrast, the range of boredom in Eastwood et al.’s definition [28] is wider than that of Russell’s model. According to their study, people who are in a low-valence state can feel boredom regardless of the level of arousal. Considering these studies, we conclude that a universally accepted definition of boredom does not exist.

Some previous studies regarding boredom categorized it as a trait, while others handled it as a state. The meaning of trait in boredom-related studies is the proneness of an individual to become bored, thus there is a difference between easily becoming bored, and being able to resist boredom. Conversely, the meaning of a state is the current state of boredom that the person is experiencing. Fahlman et al. [29] handled boredom as a trait, while Eastwood et al. [28] and Kim et al. [6] treated it as a state. In this study, we approach boredom as a state. The reason for this is that we hypothesize that, when a person feels bored, changes in their physiological signals can be identified.

Our literature review identified nine studies on boredom classification from physiological data sources as listed in Table 1. It reveals that seven studies used more than one data source; this approach of sensor fusion is a common technique to increase the detection accuracy. The median number of participants in these studies was 21, which is relatively small compared to other cases where machine learning methods are typically applied. In the individual source perspective, EEG was used by three studies, and GSR was utilized by four studies. However, to the best of our knowledge, no previous study has used both EEG and GSR data for classifying boredom.

Sanei and Chambers [30] and Ashwal and Rust [31] showed that EEG data correlates with emotion states of humans. Furthermore, GSR is related to the autonomic nervous system [32], which is also known to correlate with emotion states, thus GSR can be utilized as a potential source for emotion classification [33]. In the physiological perspective, EEG data are captured from the activity of the brain, which belongs to the nervous system together with GSR. Moreover, the analysis on the characteristics of boredom conducted by Bench and Lench [34] suggested that boredom should be associated with the increased autonomic nervous system activity. This linkage implies that a correlation may exist between boredom, EEG and GSR, but so far it has not been investigated in previous studies.

Table 2 presents the methods and the accuracy results of the previous studies that classified boredom using physiological data. Mandryk and Atkins [11], D’Mello et al. [12], Giakoumis et al. [8], and Kim et al. [6] focused on finding correlations between boredom and physiological data using statistical approaches, thus they did not generate classification models. The other reviewed boredom classification studies built classification models using machine learning algorithms and measured the performance of the models. However, these studies did not address the issue of overfitting carefully, making it difficult to guarantee the robustness of the results. Moreover, many previous studies lacked the discussion on the choice of the classification algorithms and only considered a few limited algorithms. Therefore, it is necessary to consider the potential of a wider range of classification algorithms to classify boredom. Considering these facts and shortcomings of previous studies, our study aims to produce reliable performance results based on more diverse machine learning methods.

## 3. Data Collection Methodology

This section describes the methods that we used to collect and analyze physiological data for the classification of boredom. We collected data according to the guidelines of the Declaration of Helsinki [35]. Specifically, we obtained written informed consents from the participants before the data collection, advertised data collection for inviting voluntary participants, explained to the participants that they could quit the experiment anytime they want, and provided snacks as a reward for their participation. The details of the data collection procedure are explained in the following sections.

### 3.1. Participants

We collected the EEG and GSR data from 28 Korean participants (13 males and 15 females) who were either students or staff at a university in the Republic of Korea. The participants’ ages ranged from 20 to 34, with an average age of 23.62. We collected the data in two sessions: the first session was carried out with 18 participants (6 males and 12 females), and the second session was carried out with 10 participants (7 males and 3 females). All data collection procedures were designed and executed with careful consideration of legal and ethical issues. All participants elected to join the experiment voluntarily, and they were instructed to quit the experiment at any time if they felt the urge to do so. To secure the safety of the participants, an emergency kit was prepared as a countermeasure for accidents. All collected data were anonymized and stored securely in a password-protected data storage. Finally, a data collection protocol (see Section 3.3) was designed with consideration of the participants’ legal rights.

### 3.2. Sensors

**Figure 2:**
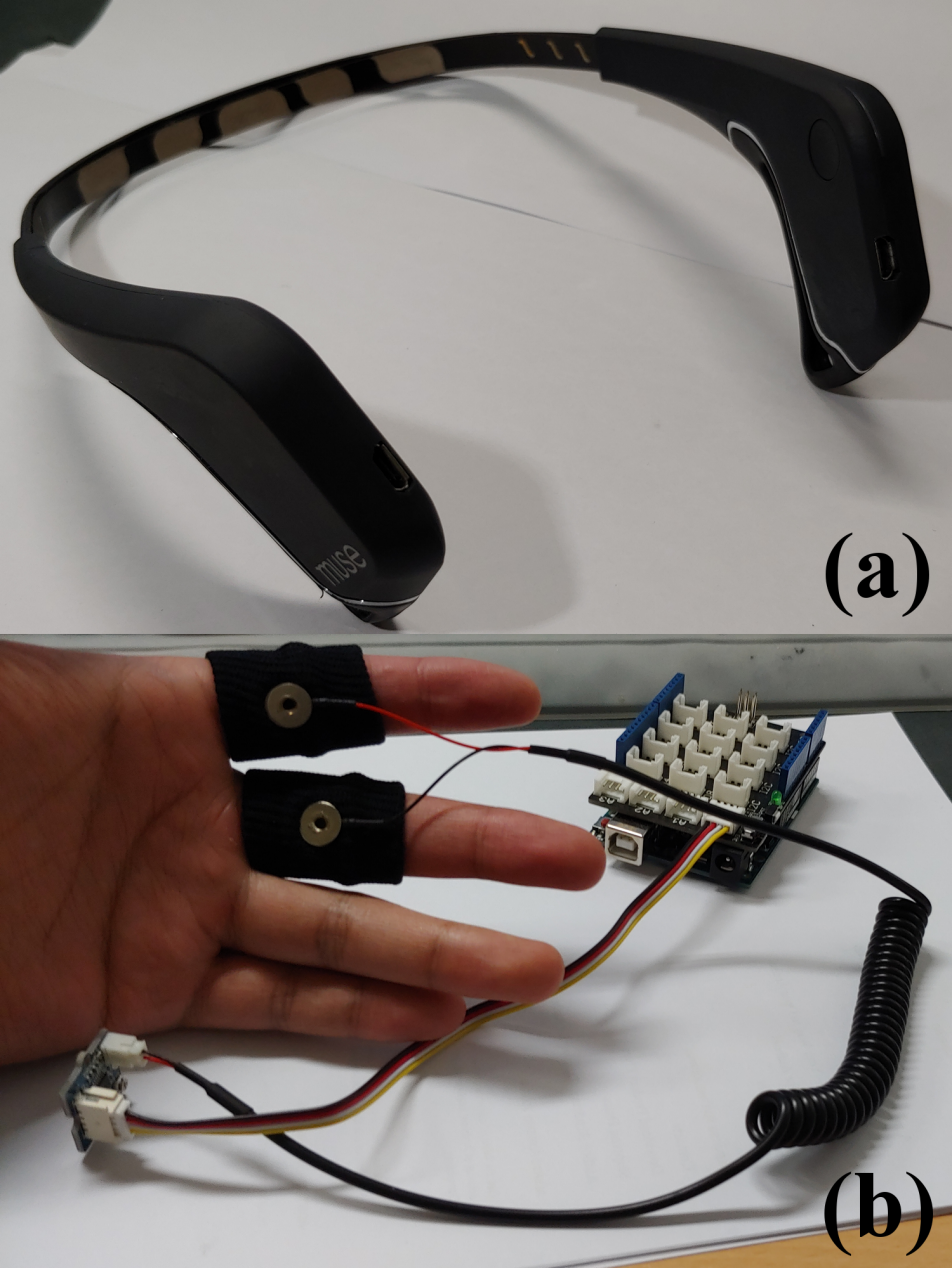
(**a**) EEG sensor, and (**b**) GSR sensor.

An EEG sensor produced by Muse [36] and a Grove GSR sensor produced by Seeed [37] were used in this study (Figure 2). The upper section of Figure 2 shows the EEG sensor, which has four electrodes. According to the instructions from the EEG sensor manufacturer, the electrodes are located at the positions FP1, FP2, TP9, and TP10 of the head [36]. The mapping of head locations was defined by Jasper [38], and it is used in neuroscience. The EEG data were captured with four electrodes; however, only the data from FP1 and FP2 were utilized. This is because TP9 and TP10 were not attached well on the participants’ heads during the data collection, which caused instability in the output data from these electrodes. The EEG sensor captured raw EEG at a 220 Hz sampling rate and provided power spectral density (PSD) values for each electrode. According to the sensor manufacturer, the types of PSD data were absolute band power (ABP), relative band power, and others [36]. The sampling rate of these data was 10 Hz. To calculate ABP, we applied the fast Fourier transform algorithm. We calculated PSD by the following EEG frequency bands [36]:

- Delta: (1–4) Hz,
- Theta: (4–8) Hz,
- Alpha: (7.5–13) Hz,
- Beta: (13–30) Hz,
- Gamma: (30–44) Hz.

The lower section of Figure 2 illustrates the Grove GSR sensor that was used in this study, along with the finger band electrodes. These electrodes were attached to the index and middle fingers of the participants. The GSR sensor captures micro voltages (MV) between the fingers using the attached electrodes. Furthermore, the sensor calculates skin resistance (SR) utilizing the MV input in ohms. The formula for calculating SR using MV is provided by the sensor manufacturer [37], and is replicated in Equation (1). The sensor was connected to an Arduino Uno device, and the captured data were transmitted to a computer at a 192 Hz sampling rate:

(1) SR=((1024+2∗MV)∗10,000)/(512−MV).

### 3.3. Protocol

**Figure 3:**
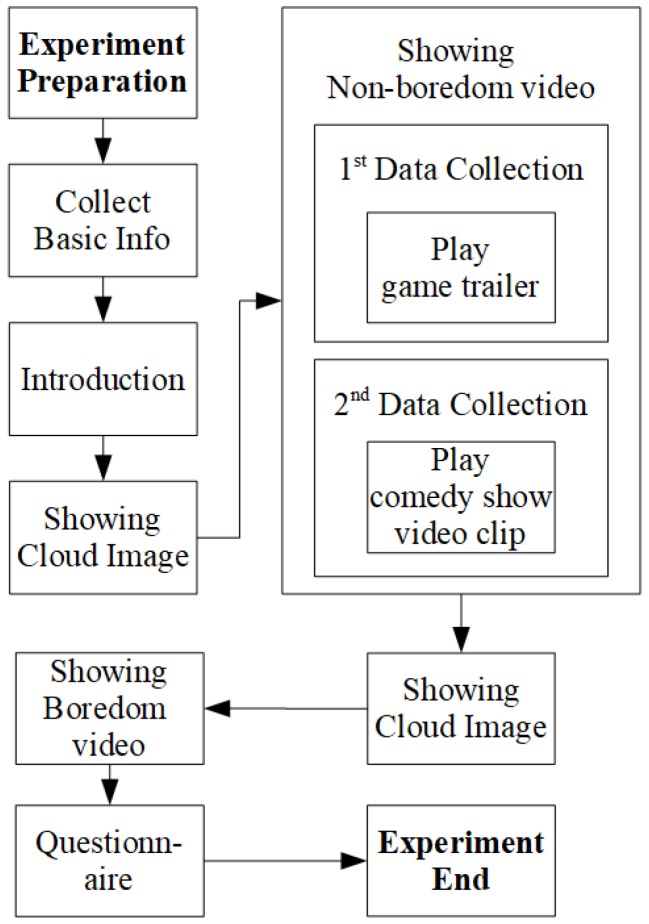
Protocol of data collection.

Figure 3 presents the protocol of data collection. In the introduction stage, the participants were presented a page showing a consent form of the experiment. The information on the consent form stated that we would use the collected data only anonymously for academic purposes. Furthermore, the participants were instructed that they could stop the experiment anytime when they feel uncomfortable.

In the stage for showing non-boredom videos, a cinematic trailer of Blizzard’s Starcraft 2: Heart of the Swarm, and a Korean comedy video clip were used to evoke non-boredom. These video clips were chosen to entertain the participants so that they would not become bored. To evoke boredom, a looping video was played in which a small circle moved slowly tracing the boundary of a bigger circle. Furthermore, to neutralize the emotion state of the participants, a cloud image from the International Affective Picture System [39] was shown for 30 seconds before showing each video stimulus. When the participants watched the videos, they were instructed to stop watching (by pressing a button) at any time they chose. Thus, each participant’s data length was different, with the shortest watching time being 7.13 s.

The data collection consisted of two sessions. The sessions were otherwise identical except for the non-boredom video stimulus (i.e., the game trailer and the comedy show clip). The reason for carrying out data collection in two sessions was to get a content-independent classification result. In other words, we wanted to see whether the change of non-boredom video has an effect on the classification. After watching the video stimuli, the participants answered a questionnaire to measure the strength of boredom that they felt. The questionnaire had two questions: one for the boredom video, and another for the non-boredom video. The questionnaires were designed to be answered on a 5-point scale, and the range of the scale was from “None” to “Very much”.

## 4. Machine Learning Methods

In this section, we describe the procedure for feature extraction and machine learning techniques for analyzing the collected data to classify boredom.

### 4.1. Window Size

As explained in Section 3.3, each participant’s data length was different because they were instructed to stop the video stimuli playback at the time they chose. The shortest data length was 7.13 s, thus only the last 7 s of each participant’s data were extracted to build the models. With the window size of 7 s, the number of samples was 56. Other window sizes, such as 1 s and 0.5 s, were also tested; however, these potentially caused overfitting because two or more samples would be generated from the same data with the same label.

### 4.2. Features

This section explains the feature extraction methods that we used for the EEG and GSR datasets. MATLAB (R2017a) was used for data analysis pertaining to feature extraction.

#### 4.2.1. EEG

Similar to our previous study [7], we extracted five EEG features: (1) ABP, (2) Normalized ABP (NABP), (3) differential entropy (DE), (4) rational asymmetry (RASM), and (5) differential asymmetry (DASM). As explained in Section 3.2, ABP is a PSD value that is produced at 10 Hz from each electrode and frequency band. NABP is a normalized value of ABP using the following equation:

(2) $$x^{\prime }=\frac{x-min(x)}{max(x)-min(x)},$$

where *x* is the original value, $x^{\prime }$ is the normalized value, and max(x) and min(x) are the maximum value and the minimum value of the dataset, respectively.

To calculate the DE, RASM, and DASM features, we used the formulae proposed by Zheng et al. [4], which are replicated in Equations (3)–(5):

(3) $$DE=\frac{1}{2}\log2\pi e\sigma^{2},$$

(4) $$DASM=DE_{\left(left\right)}-DE_{\left(right\right)},$$

(5) $$RASM=DE_{\left(left\right)}/DE_{\left(right\right)}.$$

In Equation (3), $\sigma^{2}$ is the variance of the bandpass-filtered EEG data of each frequency band, and π and *e* are constants. Furthermore, as the equations show, DASM and RASM are based on DE and utilize the electrical current asymmetry between the electrodes of the EEG sensors. Therefore, in this study, we use the asymmetry between the electrodes and the values from each electrode at the same time to train classification models.

As explained in Section 4.1, we set the window size to 7 s for feature extraction. Therefore, 70 units of ABP data and 70 units of NABP data of each electrode and frequency band, and 1540 units of EEG data of each electrode were extracted from each participant’s data. We calculated the average and the standard deviation for each 70 units of ABP and NABP data and used the results as features. In the calculation of DE, we used all 1540 units of EEG data. In more detail, we applied the bandpass filter on EEG data. Regarding the frequency range of filtering, we followed the frequency band ranges of our EEG sensor (see Section 3.2). Finally, RASM and DASM were calculated using the extracted DE values.

As a result of EEG feature extraction, we secured 40 features from ABP and NABP (five frequency bands times two electrodes times two summary statistics). Moreover, 10 features were extracted from DE (five frequency bands times two electrodes). Finally, as indicated by Equations (4) and (5), RASM and DASM utilize locational symmetry for each electrode and DE value. Thus, five features were secured from RASM and DASM (five frequency bands).

#### 4.2.2. GSR

As mentioned in Section 3.2, we used MV and SR data features from the GSR sensor. Additionally, normalization of MV with feature scaling was also performed (see Equation (2)). As a result, MV, normalized MV (NMV), and SR were secured from the GSR data. Similar to the feature extraction of the EEG data, we calculated the average and the standard deviation for each feature of the GSR data and used them as the final feature values. Consequently, six features in total were secured from the GSR data.

### 4.3. Machine Learning Model Selection

Weka, which is an open-source software for data mining that provides several machine learning algorithms, was used for building and testing the machine learning models [40]. In this study, we considered a wide range of machine learning algorithms supported by Weka as candidate algorithms. These candidates were used for initial testing to select the best algorithms for hyperparameter tuning.

Table 3 presents the evaluated algorithms and their options for training. Most of the algorithms were set to default parameters, i.e., the parameters that were preconfigured for the respective algorithms in Weka. Some algorithms (IBk, MLP, SVM) were used several times with different configurations, but these were considered as the same algorithm with different parameters. Therefore, although the number of algorithms was 19, we had 30 models in total to be trained for each dataset (EEG, GSR, and EEG-GSR combined).

IBk, which is a k-Nearest Neighbor classifier, has a parameter for getting a weight from the distance between samples. By default, no weight is assigned. In MLP, the number of layers and the number of nodes in each layer can be defined. For example, “t,i” means that the MLP has two layers, with the first layer consisting of “t” nodes and the second layer consisting of “i” nodes. Table 3 notes explain “a”, “i”, “o” and “t”. Finally, SVM provides options for selecting the SVM kernel type to be used. The MLP, IBk and SVM algorithms also have additional parameters than the ones listed in Table 3; however, we did not adjust them in this study.

### 4.4. Feature Refinement

To increase the models’ performance, we applied a feature selection algorithm provided by Weka called Wrapper Subset Evaluator (WSE). WSE was proposed by Kohavi and John [41] to find a classifier-optimized feature subset from a dataset to increase model performance. To use WSE, a searching method (forward or backward searching) is required. The target classifier information is also provided as an input to WSE. We used forward searching and the optimized feature set for each algorithm during initial testing and hyperparameter tuning. In the next section, we present the selected features that produced the highest accuracies.

## 5. Results

### 5.1. Questionnaire

**Figure 4:**
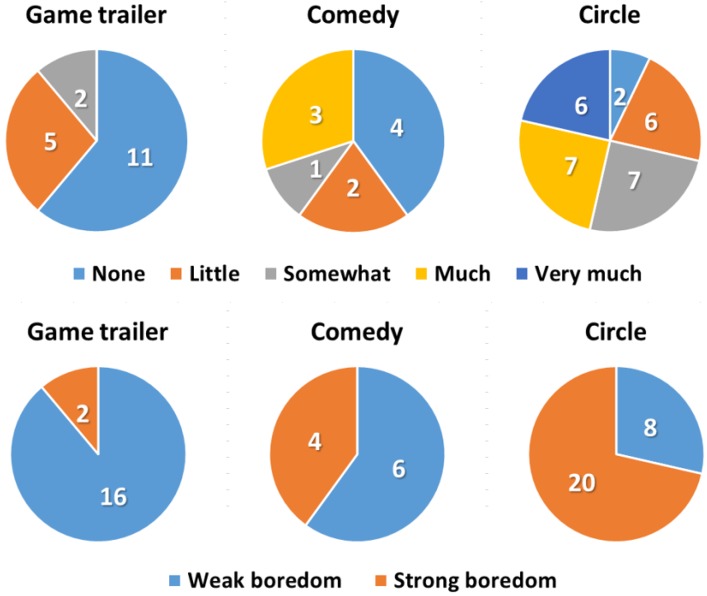
Questionnaire results (Question: How much boredom did you feel from “stimulus name”?).

We first analyzed the questionnaire results, summarized in Figure 4, to be used for labeling the datasets. We regrouped the questionnaire answers into two groups as follows: “None” and “Little” were merged into the weak boredom group, and the remaining answers were merged into the strong boredom group. The total number of questionnaire answers was 56, which comprised 28 participants’ answers for two video stimuli. The number of answers in the weak boredom group was 30, while 26 were assigned to the strong boredom group. These regrouped questionnaire results were then used for labeling the collected samples. Based on Figure 4, the game trailer and the comedy clip did not induce boredom among most participants; however, the circle video was successful in evoking boredom among the participants.

### 5.2. Initial Test for Model Selection

We performed initial testing to compare the 19 candidate algorithms and to select the best models for further analysis. Table 4 presents the top ten models for each dataset. Based on the results, we selected RF, MLP, and NB for further investigation. In particular, RF was ranked as the best algorithm for the EEG-GSR combined and EEG datasets. MLP was ranked in the top ten more than other algorithms in all datasets. We also chose NB because it has a relatively low time complexity of O(np), where *n* is the number of training samples and *p* is the number of features, whereas other algorithms with similar performance, such as IBk or J48, have time complexity of $O(n^{2})$.

### 5.3. Hyperparameter Tuning

#### 5.3.1. Random Forest

Weka’s RF API has three major hyperparameters that are related to performance: the number of features to randomly investigate, the number of trees, and the maximum depth of trees. In order to find the best hyperparameters, we trained models with all possible combinations of the parameters within predefined ranges. As a result, we trained 107,100 models and measured these performances with 10-fold cross validation. The predefined ranges of the tuning parameters were as follows:

- Number of features to randomly investigate: 1–6, default = int($\log_{2}\left(p\right)+1$),
- Number of trees: 1–100,
- Maximum depth of trees: 1–50, no limit.

Table 5 presents the top three hyperparameter combinations for the Random Forest algorithm in each dataset. To select the best hyperparameters, we established the following prioritization: (1) Accuracy, (2) Area Under the receiver operating characteristics Curve (AUC), and (3) expected classification cost. Consequently, we selected 7, 14, and 7 as the number of features, trees, and the maximum depth value, respectively, for the EEG-GSR combined dataset. In the case of EEG and GSR datasets, the default values for the number of features (6, and 3, respectively) were optimal, and 18 and 11 were selected as the number of trees and depth, respectively. The “no limit” in depth means unlimited search on each tree of random forest. Considering the expected classification cost, 11 appears to be a suitable value.

#### 5.3.2. Multilayer Perceptron

We tuned the neural network design, learning rate, and epoch parameters for MLP in the Weka API. We started by evaluating the neural network design parameters by testing all possible cases, whilst keeping the other parameters at default values. For flexible neural network design, we followed the Weka API’s MLP neural network design parameter rule (see Table 3). The general design concept of a neural network was to decrease the number of each layer’s nodes gradually, from the first layer to the last layer.

To decide the optimal learning rate and epoch values for MLP, we tested all possible combinations of these within predefined ranges. As a result, 3,600,000 models were trained and their performances were measured using 10-fold cross validation. The predefined ranges of each tuning parameter were as follows (the default value of MLP’s momentum parameter is 0.2; however, we fixed it as 0.1):

- Learning rate: 0.01–1.00 (0.01 unit),
- Epoch: 1–2000 (1 unit).

Table 6 presents the hyperparameter tuning results. To select the best hyperparameters, we established the following prioritization: (1) Accuracy, (2) AUC, and (3) low epoch. Considering the characteristics of MLP and to avoid overfitting, the learning needs to stop when the accuracy does not increase. Regarding the network design, models with three hidden layers could not be tested because WSE produced a model that contained only the label data. According to the results of Table 6, we selected “t”, 0.76, and 73 as the values of network design, learning rate, and epoch, respectively, for the EEG-GSR combined dataset. For the EEG dataset, “i”, 0.19, and 489 were found to the best values of network design, learning rate, and epoch, respectively. Finally, “t”, 0.95, and 321 were designated as the values of the three hyperparameters for the GSR dataset.

#### 5.3.3. Naïve Bayes

Table 7 presents the tuning results of the NB hyperparameters for each dataset. Weka’s NB API has two major options, which are mutually exclusive: whether to use a kernel density estimator rather than the normal distribution for numeric attributes (“Kernel” in Table 7), and whether to use a supervised discretization to process numeric attributes (“Discretization” in Table 7).

As in the parameter tuning processes for RF and MLP, we also tested all possible combinations of the NB hyperparameter options. Thus, we trained and tested nine models in total, and found that the parameters should be disabled for the EEG-GSR combined, EEG, and GSR datasets.

### 5.4. Final Performance Analysis

#### 5.4.1. Performance Measurement

A common method of evaluating a classification model’s performance is to use k-fold cross validation. However, k-fold cross validation is based on a random split of data; thus, when the validation is executed, the produced performance results can be different each time. Therefore, to obtain more reliable performance results, we measured the final models’ performance by repeating 10-fold cross validation 1000 times with different seed values. Table 8 presents the mean, maximum and minimum accuracies and AUCs produced by 1000 iterations of the 10-fold cross validation runs for each parameter combination and dataset. Considering the mean accuracy, the MLP algorithm produced the best performance in all datasets. However, when considering the mean AUC values, the RF model outperformed the MLP model on the EEG-GSR combined dataset. The last column of Table 8 reports the average computation time for each run. We measured the average time per cross validation using the last 100 iterations of 10-fold cross validation. MLP took the longest time overall, for example, it took 71 ms on the EEG-GSR combined dataset, which was about 2.7 times longer than RF and 7.5 times longer than NB. The performance on the EEG-GSR combined dataset was better than each individual data performance in all cases.

**Figure 5:**
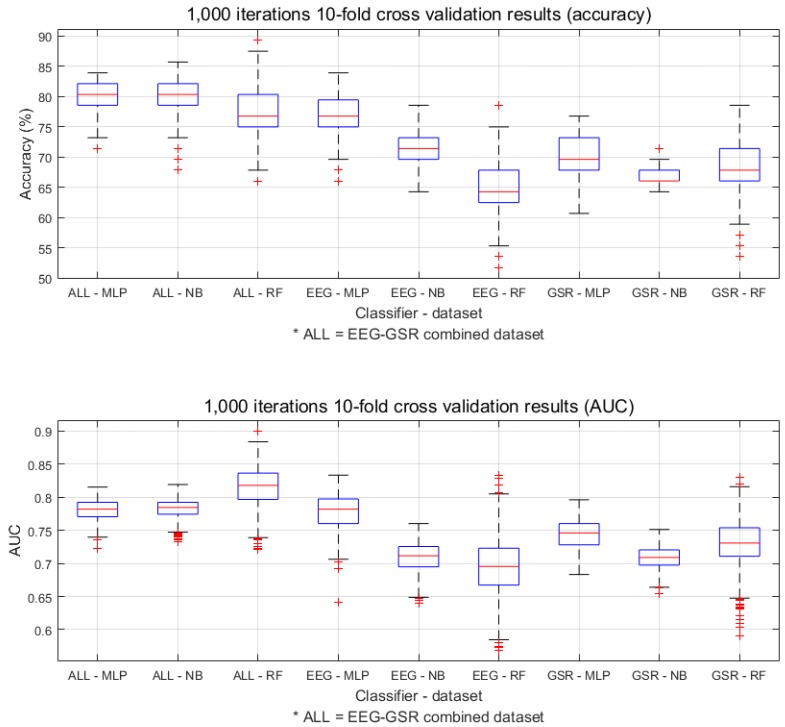
Box plots for the final performance comparison.

Figure 5 shows the models’ final performances using box plots. We observe that the MLP and NB models’ performances were more stable than that of RF in 1000 runs of 10-fold cross validation. The mean accuracies of MLP were higher than those of the other models in general. The RF model on the EEG-GSR combined dataset showed a high mean AUC but had a large variance. Muller et al. [42] defined a model’s discriminatory ability with AUC as follows: (1) excellent discrimination (AUC >= 0.90), (2) good discrimination (0.80 <= AUC < 0.90), (3) fair discrimination (0.70 <= AUC < 0.80), and (4) poor discrimination (0.60 <= AUC < 0.70). For many of our final models, the AUC was over 0.7, and thus these models can be classified as fair discrimination models.

Considering all aspects of our model performance validation, the MLP model classified boredom most reliably on all the datasets. Furthermore, the model using the EEG-GSR combined dataset showed the highest performance, while MLP on the EEG dataset and MLP on the GSR dataset ranked second and third, respectively.

#### 5.4.2. Analysis of the Selected Features

**Figure 6:**
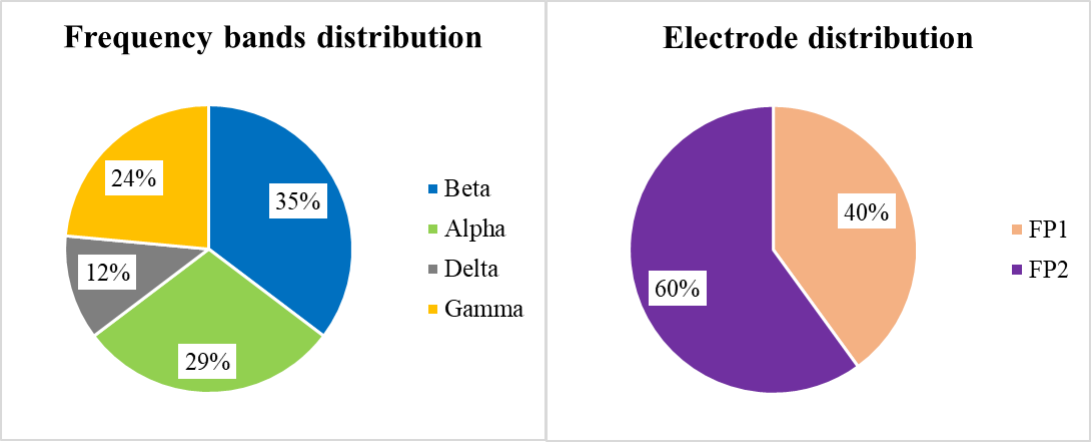
Distributions of EEG features by frequency bands and electrodes.

Table 9 presents the features that the WSE algorithm selected for each model. These results indicate that the standard deviation of MV was selected for all classifiers that used the GSR datasets. For a more detailed analysis of the selected features, we illustrate the distribution of the selected EEG features by frequency bands and electrodes in Figure 6. As the left pie chart indicates, the features related to the Alpha and Beta bands were selected more frequently than those of the Delta and Gamma bands, and the Theta band features were not selected by WSE at all. The right pie chart in Figure 6 illustrates the distribution of features by electrodes, where the distribution among FP1 and FP2 is nearly balanced; however, FP2 features were picked slightly more frequently.

**Figure 7:**
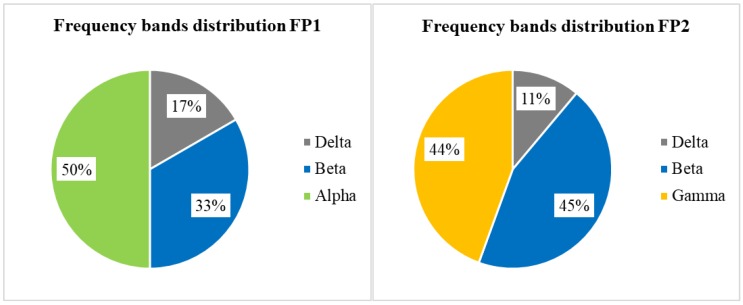
Distributions of EEG features by frequency bands for each electrode.

Figure 7 illustrates the distribution of EEG features by frequency bands for each electrode separately. We find that all selected Alpha band features are concentrated on FP1. In contrast, all selected Gamma band features occur on FP2 as the right pie chart shows.

Based on our analysis of Table 9, Figure 6 and Figure 7, we conclude that EEG and GSR show some indicators for the classification of boredom. First, the standard deviation of MV strongly correlated with boredom because this feature was selected from both GSR datasets. Second, the Gamma, Alpha and Beta bands have a strong correlation with boredom because these were selected more frequently than the Delta and Theta bands. Third, each frequency band has some correlation with a specific electrode location in boredom classification; for example, all selected Gamma band features belonged to FP2 and all selected Alpha band features belonged to FP1.

## 6. Discussion

In our experiment, the MLP models’ performances were more stable than those of the other models. Considering Table 8, the RF model for the EEG-GSR combined dataset produced a maximum accuracy of 89.29%, which is the highest of all accuracies; however, its minimum accuracy was 66.07%, which was the lowest accuracy among all the models on the same dataset. An important property of a good classification model is the robustness of performance over multiple executions. To evaluate this for the selected models, we executed 1000 iterations of 10-fold cross validation with different random seeds. Thus, training data and testing data splits were changed randomly. A good model should be able to produce good classification results for different training and testing datasets. From this aspect, the MLP models classified boredom more robustly than the other models. Furthermore, the EEG-GSR dataset’s MLP model (mean accuracy of 79.98%) outperformed the other MLP models in the aspect of mean accuracy. Therefore, our analysis suggests that MLP is generally recommended for classifying boredom from EEG and GSR.

Comparing our main results with the previous research presented in Table 2, our model’s performance is better than those of Jaques et al. [13] and lower than Jang et al. [10]. However, as Table 1 shows, previous studies did not utilize EEG and GSR for classifying boredom. Thus, a direct comparison between our model and previous studies’ performance is not reasonable. Furthermore, we collected EEG and GSR data from 28 participants whereas many previous studies, with the exception of Jang et al. [10], Jaques et al. [13], and Seo et al. [7], collected data from less than 28 participants (see Table 1); thus, our model is based on data acquired from a sufficient number of participants.

Moreover, this study executed 1000 iterations of 10-fold cross-validation on each model to reduce the effect of randomness on the results and to produce more reliable performance scores. Among previous studies, Jaques et al. [13], Jang et al. [10], and Seo et al. [7] also validated their models with 10-fold cross validation; however, they did not mention the number of repetitions of validation so we assume that cross validation was only executed once. Shen et al. [2] separated their data into training and testing sets but did not consider the random effect. Giakoumis et al. [9] validated their model with the use of leave-one-out cross validation that validates a model without random effect; however, as we mentioned above, a direct comparison of this study to our study is not reasonable because we used EEG and GSR datasets, while Giakoumis et al. [9] used ECG and GSR datasets.

We note that the proposed MLP model’s performance (79.98%) is lower than that of the model proposed in our previous study (86.73%) [7]. One of the reasons contributing to this difference is that, as we explained in the paragraph above as well as in Section 4.1 and Section 4.2.1, the experimental setting and the way we evaluated the models’ performance was modified from the previous study. These changes were made to improve the robustness and generalizability of the results. For example, in our previous study [7], we acquired multiple samples from one participant’s data by splitting them into one-second windows and used these for training; in the current study, we acquired only one sample from each participants’ dataset by increasing the window size, thus aiming to increase the independence between the samples. This can help to reduce overfitting and achieve more generalizable results. Moreover, the previous study conducted only one iteration of cross validation, whereas, in the current study, mean accuracies were recorded after 1000 iterations of 10-fold cross-validation to increase the robustness of the results. This approach provides more reliable performance scores especially in the applications where the number of available samples is relatively small as in this study. Although the aforementioned steps taken decreased the accuracy of the final model, the generalizability and reliability of the result were increased.

Another novelty of this study is the identification of correlation between EEG, GSR, and boredom through the interpretation of features. As we explained in Section 2, this correlation was not revealed by previous studies. Moreover, our findings are aligned with Bench and Lench [34]’s suggestion that boredom should increase the autonomic nervous system activity, which directly relates to EEG and GSR as data sources. In our feature refinement results, the WSE algorithm recommended the EEG and GSR features on the best performing model for increasing performance. This suggests that the combination of these data correlates with boredom. In particular, Gamma band features were selected for the combined EEG-GSR and the EEG datasets. This indicates that the Gamma band may correlate with boredom, whereas the Alpha, Beta and Delta bands have weaker correlations, and the Theta band has no correlation at all with boredom. Furthermore, the WSE algorithm selected the standard deviation of MV among the GSR features from the combined EEG-GSR and GSR datasets. Consequently, the standard deviation of MV can also be an indicator of boredom.

## 7. Conclusions

In this study, we classified boredom using features from EEG and GSR datasets that were trained and tested by 30 models based on 19 different machine learning algorithms as an initial test for finding a suitable classification algorithm. We picked MLP, RF, and NB as the most suitable candidate algorithms. After tuning the selected algorithms’ hyperparameters, we executed 1000 iterations of 10-fold cross validation with different random seed values to identify the most robust model among these. As a result, we recommended the MLP model which had a mean accuracy of 79.98% on the EEG-GSR combined dataset. Another major finding is that EEG and GSR appear to correlate with boredom, thus supporting the conclusion of Bench and Lench [34] that boredom and autonomic nervous system are linked.

Although this study produced novel contributions, there are noteworthy limitations. First, we collected physiological data from young and healthy participants. Thus, the recommended models may not be applicable to other age groups and to people with health issues. In addition, we hypothesize that emotion elicitation, and possibly also the manifestation of experienced emotions, is related to culture. We collected the data from Korean participants using non-boredom stimuli that were purposefully picked for this cultural context; therefore, the model may not be applicable to participants coming from other cultures. Regarding the protocol, we did not consider the effect of the order of showing the stimuli because the number of participants was deemed to be insufficient for dividing them into comparison groups. Finally, we used only one type of content to elicit boredom. Other types of contents may give different results about the intensity of the experienced emotion. In our future work, we aim to solve these limitations by collecting more data from a diverse group of users who are exposed to different boredom-evoking stimuli.

These results can be of use to developers building accurate affective computing systems as well as to researchers who seek to understand the physiological properties of boredom. As noted above, the current results still have limited applicability due to the experiment design that used only one type of boredom stimulus and a fairly homogeneous participant population. We plan to use diverse stimuli and extend the data collection to children, elderly people, patients suffering from different medical conditions, and participants representing other cultures to overcome these limitations.

## Figures and Tables

**Table 1:** Studies on boredom classification using physiological data.

Study	Data Source	Number of Participants
Shen et al. [2]	HR, GSR, BP, EEG	1
Mandryk and Atkins [11]	HR, GSR, Facial	12
Kim et al. [6]	Eye-tracking, EEG	16
Giakoumis et al. [9]	ECG, GSR	19
Giakoumis et al. [8]	ECG, GSR	21
Seo et al. [7]	EEG	28
D’Mello et al. [12]	Facial, Gesture	30
Jaques et al. [13]	Eye-tracking	67
Jang et al. [10]	HR, GSR, Temperature, PPG	217

HR - Heart Rate, BP - Blood pressure, ECG - Electrocardiogram, PPG - Photo Plethysmo Graphy.

**Table 2:** Methods and accuracies of previous boredom classification studies.

Study	Accuracy	Method
Jaques et al. [13]	73.0%	Random Forest
Jang et al. [10]	84.7%	Discriminant Function Analysis
Shen et al. [2]	86.3%	Support Vector Machine (SVM)
Seo et al. [7]	86.7%	k-Nearest Neighbors (kNN)
Giakoumis et al. [9]	94.2%	Linear Discriminant Analysis
Mandryk and Atkins [11], D’Mello et al. [12], Giakoumis et al. [8], and Kim et al. [6]	Not available	Statistical approaches

**Table 3:** List of algorithms used for training models.

Algorithm	Option	Algorithm	Option	Algorithm	Option
IBk	Default	Multilayer Perceptron (MLP)	t	SVM	Linear
	1/distance		i		Polynomial
	1-distance		a		Radial
Decision Stump	Default		o		Sigmoid
Decision Table	Default		t,a	LMT	Default
Hoeffding Tree	Default		t,a,o	PART	Default
J48	Default		t,i,a,o	Logistic	Default
Random Tree (RT)	Default	Random Forest (RF)	Default	Simple Logistic	Default
JRip	Default	REP Tree	Default	Zero R	Default
Naïve Bayes (NB)	Default	KStar	Default	One R	Default

**Network design parameter of MLP (Number of node per layer)**. a = (number of features + number of labels)/2, i = number of features, o = number of labels, t = number of features + number of labels, Ex) if 10 features and 2 labels are used, a, i, o, and t are 6, 10, 2, and 12, respectively (single hidden layer).

**Table 4:** Initial testing results for model selection.

EEG-GSR		EEG		GSR	
**Algorithm**	**Accuracy (%)**	**Algorithm**	**Accuracy (%)**	**Algorithm**	**Accuracy (%)**
RF	83.93	RF	80.36	MLP (t)	75.00
PART	80.36	MLP (a)	78.57	Simple Logistic	73.21
IBk	80.36	MLP (i)	78.57	MLP (a)	71.43
J48	80.36	KStar	78.57	MLP (i)	71.43
NB	80.36	MLP (o)	73.21	SVM (Radial Kernel)	71.43
RT	78.57	NB	71.43	MLP (o)	69.64
Hoeffding Tree	78.57	Hoeffding Tree	71.43	KStar	69.64
MLP (o)	76.79	MLP (t)	71.43	PART	69.64
MLP (a)	76.79	IBk	71.43	Decision Stump	69.64
MLP (t)	76.79	Logistic	69.64	J48	69.64

**Table 5:** RF hyperparameter tuning results.

	Features	Trees	Depth	Accuracy (%)	AUC
**EEG-GSR**	default (7)	14	7	87.50	0.842
	2	14	7	87.50	0.842
	3	14	7	87.50	0.842
**EEG**	default (6)	18	no limit	76.79	0.780
	default (6)	18	11	76.79	0.780
	default (6)	18	12	76.79	0.780
**GSR**	default (3)	18	no limit	76.79	0.780
	default (3)	18	11	76.79	0.780
	default (3)	18	12	76.79	0.780

**Table 6:** MLP hyperparameter tuning results.

	Layer and Node	Learning Rate	Epoch	Accuracy (%)	AUC
EEG-GSR	a	0.47	444	76.79	0.771
	i	0.90	215	82.14	0.794
	o	0.47	444	76.79	0.771
	t	0.76	73	83.93	0.765
	i, a	0.59	572	82.14	0.795
	i, o	0.49	1351	82.14	0.764
	t, i	0.91	192	76.79	0.737
EEG	a	0.70	452	76.79	0.733
	i	0.19	489	83.93	0.822
	o	0.21	654	80.36	0.751
	t	0.48	163	82.14	0.791
	i, a	0.43	405	78.57	0.706
	i, o	0.71	265	75.00	0.710
	t, i	0.99	320	78.57	0.692
GSR	a	0.44	312	75.00	0.767
	i	0.44	312	75.00	0.767
	o	0.44	312	75.00	0.767
	t	0.95	321	76.79	0.759
	i, a	0.64	1120	73.21	0.663
	i, o	0.90	231	71.43	0.642
	t, i	0.91	255	71.43	0.641

**Table 7:** NB hyperparameters’ tuning results.

	Kernel	Discretization	Accuracy (%)	AUC
**EEG-GSR**	FALSE	FALSE	82.14	0.785
	TRUE	FALSE	76.79	0.819
	FALSE	TRUE	60.71	0.569
**EEG**	FALSE	FALSE	67.86	0.653
	TRUE	FALSE	67.86	0.603
	FALSE	TRUE	53.57	0.454
**GSR**	FALSE	FALSE	69.64	0.681
	TRUE	FALSE	64.29	0.626
	FALSE	TRUE	60.71	0.569

**Table 8:** Final performance comparison—1000 runs of 10-fold cross validation.

		Accuracy (%)			AUC			Time (ms)
		Mean	Max	Min	Mean	Max	Min	Mean
**EEG-GSR**	RF	77.53	89.29	66.07	0.815	0.900	0.722	26.52
	NB	79.39	85.71	67.86	0.783	0.819	0.733	9.41
	MLP	79.98	83.93	71.43	0.781	0.815	0.723	70.98
**EEG**	RF	64.77	78.57	51.79	0.695	0.833	0.569	44.38
	NB	70.85	78.57	64.29	0.710	0.760	0.640	9.89
	MLP	77.04	83.93	66.07	0.775	0.833	0.641	251.22
**GSR**	RF	68.33	78.57	53.57	0.731	0.831	0.591	30.84
	NB	66.86	71.43	64.29	0.709	0.751	0.655	11.9
	MLP	70.03	76.79	60.71	0.744	0.796	0.683	163.49

**Table 9:** Selected features by WSE in each model.

	EEG-GSR	EEG	GSR
MLP	ABP Delta FP1 std	ABP Beta FP2 mean	MV std NMV mean
	ABP Gamma FP2 mean	ABP Gamma FP2 mean	
	NABP Delta FP2 mean	NABP Alpha FP1 std	
	MV std	DASM Alpha	
NB	ABP Beta FP2 mean	NABP Alpha FP1 std DE Beta FP1	MV std NMV std
	ABP Gamma FP2 mean		
	NABP Alpha FP1 mean		
	NABP Beta FP1 mean		
	MV std		
RF	NABP Beta FP2 mean MV std	ABP Gamma FP2 mean	MV std SR mean
		NABP Beta FP2 mean	
		Alpha RASM	

## References

[1] Picard R.W. (1995). Affective Computing.

[2] Shen L., Wang M., Shen R. (2009). Affective e-Learning: Using “Emotional” Data to Improve Learning in Pervasive Learning Environment Related Work and the Pervasive e-Learning Platform. Educ. Technol. Soc..

[3] Zheng W.L., Lu B.L. (2015). Investigating Critical Frequency Bands and Channels for EEG-Based Emotion Recognition with Deep Neural Networks. IEEE Trans. Auton. Mental Dev..

[4] Zheng W.L., Zhu J.Y., Lu B.L. (2017). Identifying Stable Patterns over Time for Emotion Recognition from EEG. IEEE Trans. Affect. Comput..

[5] Kim J.J., Fesenmaier D.R. (2015). Measuring emotions in real time: Implications for tourism experience design. J. Travel Res..

[6] Kim J., Seo J., Laine T.H. (2018). Detecting Boredom from Eye Gaze and EEG. Biomed. Signal Process. Control.

[7] Seo J., Laine T.H., Sohn K.A. (2019). Machine learning approaches for boredom classification using eeg. J. Ambient Intell. Human. Comput..

[8] Giakoumis D., Vogiannou A., Kosunen I., Moustakas K., Tzovaras D., Hassapis G. Identifying Psychophysiological Correlates of Boredom and Negative Mood Induced During HCI. Proceedings of the 1st International Workshop on Bio-Inspired Human-Machine Interfaces and Healthcare Applications.

[9] Giakoumis D., Tzovaras D., Moustakas K., Hassapis F. (2011). Automatic recognition of boredom in video games using novel biosignal moment-based features. IEEE Trans. Affect. Comput..

[10] Jang E.H., Park B.J., Park M.S., Kim S.H., Sohn J.H. (2015). Analysis of physiological signals for recognition of boredom, pain, and surprise emotions. J. Physiol. Anthropol..

[11] Mandryk R.L., Atkins M.S. (2007). A fuzzy physiological approach for continuously modeling emotion during interaction with play technologies. Int. J. Hum. Comput. Stud..

[12] Sidney K.D., Craig S.D., Gholson B., Franklin S., Picard R., Graesser A.C. Integrating Affect Sensors in an Intelligent Tutoring System. Proceedings of the Affective Interactions: The Computer in the Affective Loop Workshop at 2005 International Conference on Intelligent User Interfaces.

[13] Jaques N., Conati C., Harley J.M., Azevedo R. (2014). Predicting affect from gaze data during interaction with an intelligent tutoring system. Lecture Notes in Computer Science.

[14] Thompson W.T., Lopez N., Hickey P., DaLuz C., Caldwell J.L., Tvaryanas A.P. (2006). Effects of Shift Work and Sustained Operations: Operator Performance in Remotely Piloted Aircraft (Op-Repair).

[15] Britton A., Shipley M.J. (2010). Bored to death?. Int. J. Epidemiol..

[16] Kanevsky L.S. (1994). A comparative study of children’s learning in the zone of proximal development. Eur. J. High Ab..

[17] Oroujlou N., Vahedi M. (2011). Motivation, attitude, and language learning. Proc. Soc. Behav. Sci..

[18] Sottilare R., Goldberg B. (2012). Designing adaptive computer-based tutoring systems to accelerate learning and facilitate retention. J. Cogn. Technol.

[19] Yeager D.S., Henderson M.D., Paunesku D., Walton G.M., D’Mello S., Spitzer B.J., Duckworth A.L. (2014). Boring but important: A self-transcendent purpose for learning fosters academic self-regulation. J. Personal. Soc. Psychol..

[20] Baker R., D’Mello S., Rodrigo M., Graesser A. (2010). Better to be frustrated than bored: The incidence and persistence of affect during interactions with three different computer-based learning environments. Int. J. Hum. Comput. Stud..

[21] Fagerberg P., Ståhl A., Höök K. (2004). EMoto: Emotionally engaging interaction. Pers. Ubiquitous Comput..

[22] Feldman L. (1995). Variations in the circumplex structure of mood. Personal. Soc. Psychol. Bull..

[23] Li M., Lu B.-L. Emotion classification based on gamma-band EEG. Proceedings of the 2009 Annual International Conference of the IEEE Engineering in Medicine and Biology Society.

[24] Lin Y.P., Wang C.H., Jung T.P., Wu T.L., Jeng S.K., Duann J.R., Chen J.H. (2010). EEG-based emotion recognition in music listening. IEEE Trans. Biomed. Eng..

[25] Shen L., Leon E., Callaghan V., Shen R. Exploratory research on an Affective e-Learning Model. Proceedings of the Workshop on Blended Learning.

[26] Russell J.A. (1980). A circumplex model of affect. J. Personal. Soc. Psychol..

[27] Vogel-Walcutt J.J., Fiorella L., Carper T., Schatz S. (2012). The definition, assessment, and mitigation of state boredom within educational settings: A comprehensive review. Educ. Psychol. Rev..

[28] Eastwood J.D., Frischen A., Fenske M.J., Smilek D. (2012). The Unengaged Mind: Defining Boredom in Terms of Attention. Perspect. Psycholog. Sci..

[29] Fahlman S.A., Mercer-Lynn K.B., Flora D.B., Eastwood J.D. (2013). Development and Validation of the Multidimensional State Boredom Scale. Assessment.

[30] Sanei S., Chambers J.A. (2013). EEG Signal Processing.

[31] Ashwal S., Rust R. (2003). Child neurology in the 20th century. Pediatr. Res..

[32] Martini F.H., Bartholomew E.F. (2002). Essentials of Anatomy and Physiology.

[33] Carlson N.R. (2012). Physiology of Behavior.

[34] Bench S.W., Lench H.C. (2013). On the function of boredom. Behav. Sci..

[35] World Medical Association (2001). World Medical Association Declaration of Helsinki. Ethical principles for medical research involving human subjects. Bull. World Health Organ..

[36] MUSE MUSE TM Headband. http://www.choosemuse.com/.

[37] Seeed Grove—GSR Sensor. http://wiki.seeedstudio.com/Grove-GSR_Sensor/.

[38] Jasper H. (1958). Report of the committee on methods of clinical examination in electroencephalography: 1957. Electroencephalogr. Clin. Neurophysiol..

[39] Lang P.J., Bradley M.M., Cuthbert B.N. (2008). International Affective Picture System (IAPS): Affective Ratings of Pictures and Instruction Manual.

[40] Hall M., Frank E., Holmes G., Pfahringer B., Reutemann P., Witten I.H. (2009). The WEKA data mining software: An update. ACM SIGKDD Explor. Newsl..

[41] Kohavi R., John G.H. (1997). Wrappers for feature subset selection. Artif. Intell..

[42] Muller M.P., Tomlinson G., Marrie T.J., Tang P., McGeer A., Low D.E., Detsky A.S., Gold W.L. (2005). Can Routine Laboratory Tests Discriminate between Severe Acute Respiratory Syndrome and Other Causes of Community-Acquired Pneumonia?. Clin. Infect. Dis..

